# Effect of Isosporiasis Prevention with Toltrazuril on Long-Term Pig Performance

**DOI:** 10.1100/2012/486324

**Published:** 2012-04-01

**Authors:** K. Rypula, M. Porowski, J. Kaba, M. Gorczykowski, A. Deniz

**Affiliations:** ^1^Division of Infectious Diseases and Veterinary Administration, Department of Epizootiology with Clinic of Birds and Exotic Animals, Faculty of Veterinary Medicine, Wrocław University of Environmental and Life Sciences, Grunwaldzki Square 45, 50-366 Wrocław, Poland; ^2^Division of Infectious Diseases and Epidemiology, Department of Large Animal Diseases, Faculty of Veterinary Medicine, Warsaw University of Life Sciences—SGGW, Nowoursynowska 159c, 02-776 Warsaw, Poland; ^3^Divison of Parasitology, Department of Internal Diseases with Clinic of Horses, Dogs and Cats, Faculty of Veterinary Medicine, Agricultural University of Wrocław, Poland; ^4^Bayer HealthCare Animal Health, Leverkusen, Germany

## Abstract

The efficacy of toltrazuril treatment was assessed in two experiments in Polish swine herds. Experiment 1 included a toltrazuril treatment group, Group A (*n* = 410), and untreated control, Group B (*n* = 386). Time to sale in Group A was 108 days versus 120 days for Group B, with average body weights at sale of 114.2 kg and 108.8 kg, respectively (*P* < 0.05). In experiment 2, the health status and body weight gain of 238 piglets treated with toltrazuril (Group D) were compared to 235 untreated piglets (Group K). A similar difference was observed in average body weights of slaughtered animals, being on average 104 kg in Group D and 101 kg in Group K (*P* < 0.01). Animals from Group D were slaughtered 5 days earlier than animals from Group K (day 166 versus day 171). Data from clinical trials suggest treatment of coccidiosis with toltrazuril offering potential for improved animal welfare and yields, however this has remained unproven in field conditions in large swine production facilities. The present study confirms the efficacy of toltrazuril treatment when used in the field and the subsequent positive impact on time to weaning, time to market, and on weight gain at all time points.

## 1. Introduction

Diarrhoea remains a significant, though often underappreciated, issue in both economic and welfare terms on many swine farms worldwide. The most common cause of severe diarrhoea in pigs aged 5–20 days is coccidiosis, caused by infection by the near ubiquitous Eimeriidae coccidium *Isospora suis* [1–3], which has been found in over 75% of farms examined in Ireland, Germany, the Netherlands, Greece, Austria, and Switzerland [4, 5]. A recent study into *I. suis* prevalence in Poland found that 90% of farms tested positive [4]. 

The *I. suis* parasite lifecycle has prepatent and patent phases; the prepatent usually lasts 5–7 days, and the patent lasts 5–16 days. Along with the characteristic 5–8 days of creamy yellow diarrhoea, serious damage to the mucosal crypts and villi of suckling piglets due to *I. suis* has previously been reported [1, 3]. The rupture of cells of the villi of gut mucosa during infection causes fibrinous enteritis and long-term damage to the gut, leaving piglets more susceptible to other infections, such as *E. coli and Clostridium perfringens* [7, 8]. Maturity of mucosa villous at weaning is of special importance in terms of postweaning feeding performance [9, 10]. Damage to the gut can also have a significant impact on the long-term growth and wellbeing of the piglet, causing decreases in weight at weaning of as much as 1 kg [6], even in asymptomatic pigs [11].

As *I. suis* infection is spread through faecal matter, hygiene is a key component of coccidiosis prevention. In theory, clearing farrowing pens and a programme of thorough, ongoing cleansing with an effective oocysticide may to some extent control the reappearance of coccidiosis, but not totally [12]. However, as *I. suis* infection is so prevalent, this is, in practice, not effective strategy.

Treatment with sulphonamides and diclazuril in experimental studies has not proven effective in managing coccidiosis, but treatment simplicity and efficacy were recently significantly improved by the approval of toltrazuril (Baycox 5%, Bayer Animal Health) for the treatment of coccidiosis in swine. Individual treatment of piglets around day 4 of life with 0.4 mL/kg toltrazuril has proven to be the most effective treatment option available [5, 6].

A number of studies have reported that treatment with toltrazuril can improve the health of the gut and increased weight gain [6, 8, 11], reduce use of antibiotics [13] and rate of diarrhoea in early life [8], and improve postweaning feed conversion ratios [9, 10].

The present study sets out to investigate the efficacy of toltrazuril treatment in swine herds in field conditions in Poland to ascertain whether the efficacy reported in clinical studies can be replicated in the field, particularly in relation to increased weight gain, reduced need for veterinary intervention and faster time to market.

## 2. Materials and Methods

### 2.1. Experiment 1

The therapeutic efficacy of a single oral treatment with toltrazuril (Baycox 5%, Bayer Animal Health) (0.4 mL/kg BW, equivalent to 20 mg toltrazuril/kg BW) was examined on two pig farms in Lower Silesia and Mazowia voividships, Poland.

Farm 1 operated a closed, continuous flow with all-in-all-out (AIAO) system of production cycle with a basic herd of 130 hybrid Polish Landrace x Polish Large White sows. Study groups comprising 10 sows were created twice a week during the study period. All welfare conditions in the labour sections as well as in the breeding and fattening sections were satisfactory. A history of *I. suis* infection was confirmed on Farm 1. 

Farm 2 also operated a closed, continuous flow with AIAO system of production cycle, with a basic herd of 450 hybrid PIC sows. Study groups of 20 sows were created once a week during the study period. All welfare conditions in the labour sections as well as in the breeding and fattening sections were satisfactory. All weaning piglets were sold on. A history of *I. Suis* infection was confirmed on Farm 2.

**Figure 1:**
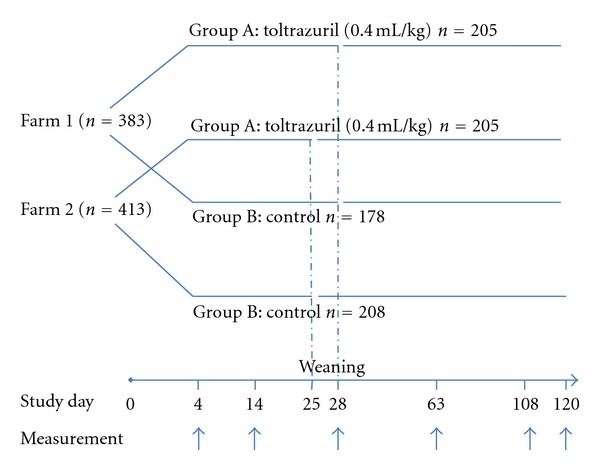
Experiment 1: Study design.

The study was conducted on 383 pigs from Farm 1 and on 413 pigs from Farm 2 based on simple randomisation. On both farms, the group treated with toltrazuril was labelled Group A (Farm 1, *n* = 205; Farm 2, *n* = 205) and the nontreated control group as Group B (Farm 1, *n* = 178; Farm 2, *n* = 208) (Figure 1). Selection criteria were based on the age of piglets and number of piglets in the litter.

Treatment efficacy assessment was based on weight gain from day 4 (first day of toltrazuril administration) through to day 14—defined as the first stage preweaning period—and from day 14 to the day of weaning—the second stage preweaning period. Piglets were weaned when 28 and 25 days old on farm 1 and 2, respectively. In addition, the frequency of medical interventions (mainly antibiotic injections) required for diarrhoea control was measured. Prior to treatment with toltrazuril, the presence of *I. suis* infection was confirmed by parasitological examination of 10% of piglet faeces in all experimental groups. Oocysts were confirmed in all collected samples.

### 2.2. Experiment 2

The second experiment was carried out on a production facility in Mazovia viovodship, Poland with a closed, AIAO production cycle and a basic herd of 1,700 hybrid PIC sows and 10 hybrid Polish Landrace x Polish Large White boars. Study groups comprising 90 sows were created once a week during the production cycle. All welfare conditions in the labour sections as well as in the breeding and fattening sections were satisfactory.

**Figure 2:**
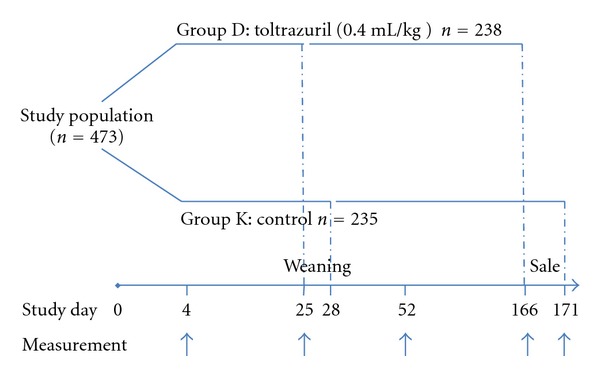
Experiment 2: Study design.

The study evaluated the health status and body weight gain of 238 piglets treated with a single dose of toltrazuril (0.4 mL/kg BW, equivalent to 20 mg toltrazuril/kg BW) (Group D) at day 4 of life compared to 235 untreated piglets in the control group (Group K) (Figure 2). The choice of the piglets was conducted as in experiment 1.

Assessments of health status and body weight gain were made on the 4th day of life (start of treatment), on the day of weaning, when moving from the breeding sector to the fattening sector, and finally on the day of sale.

Statistical calculations in both experiments were carried out with Mann-Whitney *U* test and *χ* ^2^ (chi-squared) test with the use of Statistica v. 6.1 software.

## 3. Results

### 3.1. Experiment 1

Body weight gain of the piglets in Group A (treatment group), between day 4 and day 14, was 2070 g and 1845 g in Farms 1 and 2, respectively. Additional body weight gain in Farms 1 and 2 in the second phase of weaning (day 14 to weaning) was 3582 g and 2580 g, respectively.

Piglets in Group B (control group) gained 1477 g on Farm 1 and 978 g on Farm 2 between day 4 and 14, and additional 2737 g and 2345 g, respectively, between day 14 and weaning.

In the time to weaning, piglets in Group A required 6 medical interventions in both Farms 1 and 2, compared to 13 and 18 in Farms 1 and 2, respectively, for those in Group B.

Piglets on Farm 1 were also followed through to sale to ascertain the impact of treatment on long-term growth. At transfer from weaning to a feeding group at day 63, average body weight was 21300 g for Group A versus 20500 g in Group B. Time to sale in Group A was 108 days for Group A versus 120 days for Group B, with average body weights at sale of 114200 g and 108800 g (*P* < 0.05), respectively (Table 1).

### 3.2. Experiment 2

Piglets in experiment 2 of the study were assessed at days 4, 25, 52, 166, and 171. Those piglets in treatment group D (*n* = 238 at birth, *n* = 210 at weaning) weaned on average at 25.6 days, while those in the control group K (*n* = 235 at birth, *n* = 194 at weaning) weaned on average at 28 days.

There was a statistically significant difference (*P* = 0.03) in relation to average daily weight gain between Group D (221 g) and Group K (204 g) at day 25, and a statistically significant difference was also observed between Groups D and K in relation to reports of diarrhoea prior to weaning—4 versus 22 cases, respectively (*P* < 0.01). Body weight at weaning and cases of digestive disorders or death showed no statistical difference between groups.

After moving animals to the breeding sector on day 25, average body weight of the animals in the group receiving the toltrazuril was 6180 g and was greater than the body weight of the piglets in Group K (*P* < 0.01). After weaning, animals were kept in their groups for fattening until day 52. Average body weight of animals transferred to the fattening sector was 31000 g and 27900 g, respectively, for Groups D and K. A similar difference was observed in average body weights of slaughtered animals, being on average 104000 g in Group D and 101000 g in Group K (Table 2). Animals from Group D were slaughtered 5 days earlier than animals from Group K (day 166 versus day 171).

## 4. Discussion

Alongside the obvious short-term impact of diarrhoeal disease, coccidiosis infection is responsible for significant damage to the gut of piglets, regardless of whether or not they are symptomatic. This damage can have serious long-term effects on their health and growth, leading to significant losses for the pig producer.

A number of experimental trials have demonstrated the impact of toltrazuril on key production criteria including daily weight gain, where treatment-related increases in weight gain have been recorded at 37 g/day to day 28 [6], 25 g/day to weaning [11], and 14 g/day between days 21 and 105 [9], and toltrazuril treatment-related feed conversion rates in weaner-finisher herds of 2.41 versus 2.63 [10] and 1.6 versus 1.77 [9]. Treatment with toltrazuril has also been shown to increase body weight at weaning by 670 g (*P* < 0.05) [6] and 326.6 g (*P* = 0.044) [8] compared to placebo.

While not directly investigating daily weight gains or feed conversion rates, the present study has confirmed the significant improvements in yield suggested by trial results are indeed replicable in the “real world”, with treatment-related weight gain of 1412 g and 350 g at weaning and 5400 g and 3000 g, respectively, for experiments 1 and 2 at time to market. It was also notable that all treatment groups reached weaning (3 days) and market weight (between 5 and 12 days) substantially earlier than the untreated controls.

Cases of diarrhoea were also significantly reduced in treated groups, reflecting the previously reported findings of McOrist and others [9] and Westphal and others [8]. The absence of significant reductions in cases of digestive disorders and death among groups in this study is likely to reflect the already low mortality associated with coccidiosis infection along with the multiplicity of infections (Porcine Respiratory Disease Complex) affecting piglets during the weaning phase in a commercial swine production facility.

The results of this study, performed in field conditions, suggest that the use of a single application of toltrazuril is likely to produce results similar to those noted in experimental trials. These results support the view that, in real-world conditions, metaphylaxis with toltrazuril is extremely effective at managing the impact of *I. suis* infection on the health and growth of piglets, leading to substantial improvements in weight gain that continue over the lifetime of the pig, bringing pigs to market weight substantially faster and at a greater weight.

## Figures and Tables

**Table 1:** Experiment 1: Mean piglet body weight.

		Piglet body weight					
		Day 4	Day 14	Day 28	Day 63	Day 108	Day 120
Farm 1	Group A	2093 g ± 390	4163 g* ± 854	7745 g ± 1843	21300 g* ± 1351	114200 g* ± 3856	—
	Group B	2125 g ± 420	3602 *g* ± 571	6333 g ± 933	20500 g ± 1381	—	108800 g ± 3036
Farm 2	Group A	2065 g ± 413	3910 g* ± 879	6490 g* ± 1337	—	—	—
	Group B	2062 g ± 374	3040 g ± 440	5385 g ± 1118	—	—	—

*P < 0.05.

**Table 2:** Experiment 2: Mean piglet body weight.

	Piglet body weight				
	Day 4	Day 25^a^/28^b^	Day 52	Day 166	Day 171
Group D	1530 g ± 103	6180 g* ± 271	31000 g* ± 1068	104000 g* ± 7026	—
Group K	1540 g ± 97	5830 g ± 245	27900 g ± 1407	—	101000 g ± 7251

*P < 0.01.

^a^Day of weaned piglets in group D.

^b^Day of weaned piglets in group K.
